# Knowledge before belief ascription? Yes and no (depending on the type of “knowledge” under consideration)

**DOI:** 10.3389/fpsyg.2022.988754

**Published:** 2022-09-12

**Authors:** Hannes Rakoczy, Marina Proft

**Affiliations:** Department of Developmental Psychology, University of Göttingen, Göttingen, Germany

**Keywords:** knowledge, belief, factive, Theory of Mind, aspectuality, mental states

In an influential paper, Jonathan Phillips et al. have recently presented a fascinating and provocative big picture that challenges foundational assumptions of traditional Theory of Mind research (Phillips et al., 2021). Conceptually, this big picture is built around the main claim that ascription of knowledge is primary relative to ascription of belief. The primary form of Theory of Mind (ToM) thus is so-called factive ToM that centers around knowledge-related mental states that are true rather than meta-representational ToM that centers around subjective epistemic states like belief that may or may not be true (Nagel, 2017; Phillips and Norby, 2021). Empirically, Phillips and colleagues build on converging findings from different areas: Ample research in developmental psychology shows that children track who has had informational access to events (and thus knows about the events) before they keep track of others' potentially false beliefs (e.g., Perner and Roessler, 2012). Many studies from comparative psychology have found evidence that non-human great apes keep track of others' perceptual and informational access while there is no convincing evidence that they keep track of others' beliefs (Call and Tomasello, 2008; Martin and Santos, 2016; Horschler et al., 2020). And, work from cognitive psychology and experimental philosophy suggests that adults are faster, for example, to judge what others know than to judge what they believe (Phillips et al., 2018).

In this commentary, we would like to critically evaluate and friendly amend the claims put forward by Phillips and colleagues. Conceptually, while we agree that some form of factive Theory of Mind is primary, we would like to raise doubts whether this primary factive ToM already involves full-fledged knowledge ascription. Empirically, we will point to potential test cases that are suitable to test Phillips and colleagues' account against the friendly amendment proposed here.

## Is knowledge ascription really primary relative to belief ascription?

We agree that the empirical findings reviewed by Phillips and colleagues do make a strong case for the conjecture that some form of factive ToM is indeed (phylogenetically, ontogenetically, and cognitively) primary. But we suspect that this claim, in unqualified form, may be somewhat incomplete and misleading. There is not necessarily one unitary form of factive ToM, and one notion of “knowledge“ in play across development and evolution, and perhaps not even in adults' Theory of Mind. This suspicion builds on several foundations: First, from an empirical point of view there have been, as highlighted by the authors, characteristic U-shaped developmental curves in some tasks of factive ToM—often a reliable indicator that different underlying processes are in play (Karmiloff-Smith, 1992). Second, from a theoretical point of view, conceptual change and dual process approaches to ToM and other forms of social cognition have highlighted the possibility of more complex developmental trajectories such that earlier and more basic forms of a conceptual competence may be supplemented and superseded by later and more sophisticated refinements (e.g., Perner, 1991; Apperly and Butterfill, 2009).

For the case of factive ToM, it may be that there is a basic and primitive notion of “knowledge“ in place early in ontogeny (and perhaps phylogenetically more ancient) that shares some of the essential features of our mature “knowledge” concept: “knowledge” in this broad sense, as emphasized by the authors, is factive, not modality-specific, and allows for representations of egocentric ignorance. For this basic concept, the slogan “knowledge before belief” may well be true. However, this basic concept need not yet be our mature notion of knowledge proper and thus this basic form of factive ToM may fall crucially short of our adult form of factive ToM. Why? Because essential elements of our mature concept of knowledge are still missing: First of all, while so-called Gettier cases and other considerations make clear that knowledge does not reduce to justified true belief (one can have justified true beliefs that still do not amount to knowledge; Gettier, 1963), according to many accounts knowledge at least presupposes justified true belief. Correspondingly, ascription of knowledge would thus presuppose ascription of belief. Now, we understand that this is one of the very traditional assumptions that Phillips and colleagues challenge, and given space restrictions we will not focus on it here any further (see, e.g., Rose and Schaffer, 2013). But there is a second crucial aspect of knowledge proper that is missing from the basic notion: knowledge proper is *aspectual*, and consequently reports of knowledge proper are *intensional*, while neither seems to be the case of basic knowledge and reports of it. Knowledge proper is aspectual in the following sense: whether or not someone knows something depends on how, under which aspects, she has had informational access to a given scene. Suppose Eve has seen Clark Kent enter the house. Does she know that Superman is in the house? It depends. If she knows about the identity Clark Kent = Superman, she does, otherwise she does not. Consequently, knowledge ascription is intensional in the sense that the substitution of co-referential terms is not necessarily truth-value preserving: “Eve knows that Clark Kent is in the house” does not imply “Eve knows that Superman is in the house”.

Now, from the point of view of cognitive development, much research suggests that children's appreciation of the aspectuality of propositional attitudes (and the intensionality of propositional attitude reports) develops in protracted ways not before the age of four (e.g., Apperly and Robinson, 1998; Rakoczy et al., 2015; Proft et al., 2019). In fact, recent studies suggest that around age four children undergo a fundamental and coherent cognitive revolution: they acquire a solid meta-representational notion of propositional attitudes that allows them to ascribe subjective aspectual representations that may or may not be accurate: Children come to solve false belief tasks that require belief ascription at the same time as tasks that require an understanding of aspectuality, and there is strong convergence/correlation between these different tasks (Rakoczy et al., 2015; Rakoczy, 2017).

These considerations thus evoke a somewhat modified picture of the developmental course of factive Theory of Mind: Some form of factive ToM, indeed, comes first (developmentally and phylogenetically). In this primary stage, subjects track agents' cognitive relations to the world that display some of the essential signatures of knowledge proper (factive; not modality-specific; allow for representations of egocentric ignorance). Various approaches in ToM research over the last years have aimed at describing this basic form of knowledge-like relations, for example in terms of “cognitive connections” (Flavell, 1988), “registration” (Apperly and Butterfill, 2009), “experiential records” (Perner and Roessler, 2012) or “awareness relations” (Martin and Santos, 2016). While differing in focus and details, all these accounts converge in stressing one crucial point: this early form of factive ToM allows observer to keep track of *what* others have or have not witnessed and, in this sense, what they do or do not know. It allows, in other words, so-called “Level I” perspective-taking (Flavell, 1977): understanding *what* others see. But this early form of factive ToM still falls short of knowledge ascription proper because it lacks an appreciation of the essential aspectuality of propositional attitudes in general and of knowledge in particular. In other words, it does not yet allow for “Level II” perspective-taking: understanding *how* different agents may represent a given scene (Low and Watts, 2013; Fizke et al., 2017; Oktay-Gür et al., 2018).

Only later, around age 4, do children then develop the new meta-representational framework of propositional attitudes that goes beyond basic factive ToM. Once they have this framework and thus an understanding of aspectuality at hand, they can extend their initial and primary factive ToM to acquire the mature concept of knowledge (as at least presupposing true, justified belief, where belief is necessarily aspectual). So, while basic knowledge ascription indeed precedes belief ascription, full-blown attribution of aspectual knowledge develops in tandem with belief attribution. Or in other words: basic factive ToM precedes full-blown meta-representational ToM but full-blown factive ToM does not (since it is itself a part of full-blown meta-representational ToM).

## Empirical outlook

This slightly modified picture raises many interesting new empirical questions, and makes competing predictions relative to the picture put forward by Phillips and colleagues:

From developmental and comparative perspectives, the modified picture would predict that “knowledge before belief” only applies for a circumscribed set of knowledge-related situations: those in which knowledge ascription does not require sensitivity to the aspectuality of knowledge (does not require distinguishing, for example, “Does she know that Clark Kent is in the house?” vs. “Does she know that Superman is in the house?”) and is limited to Level I perspective-taking. Young children before the age of four and non-human primates should be able to solve such non-aspectual knowledge ascription problems. But only older children from around age four, once they have acquired the full-fledged conceptual apparatus of meta-representation, should be able to handle aspectual knowledge ascription.

Regarding adult functioning, the most fundamental question is: Do adults operate with one unitary factive ToM, as Phillips and colleagues assume? Or are there two kinds of factive ToM throughout the lifespan, as our modified picture suggests? In particular, does the more basic version remain in operation in adulthood, perhaps even as the default mode, that reveals itself under conditions of speeded responses, limited cognitive resources etc.? If the latter were true, specific performance patterns should be found. First, results such that adults are faster at knowledge ascription than at belief ascription (as found in Phillips et al., 2018) should be restricted to designs where knowledge ascription does not require any considerations of aspectuality. In such designs (as they were used in Phillips et al., 2018), subjects can make use of their primordial (non-aspectual) factive ToM in knowledge ascription, but have to use their full-fledged (aspectual) ToM in belief ascription. However, in new cases in which knowledge ascription is potentially aspectual (“Does Eve know that Superman is in the house?”), the speed difference between knowledge and belief ascription should vanish since both now require full-fledged (aspectual) ToM.

Second, and relatedly, fast factive ToM should have characteristic signature limits to do with the lacking appreciation of aspectuality (Apperly and Butterfill, 2009; Low et al., 2016): Subjects under speeded conditions (or in dual task formats in which their central cognitive resources are taxed) should be unable to systematically distinguish between “Eve knows that Clark Kent is the house” (true) and “Eve knows that Superman is in the house” (possibly true, possibly false). No such signature limits should be expected, in contrast, under reflective conditions in which subjects can use their full-fledged and mature factive ToM. Interestingly, these hypothetical developmental and adult performance patterns would correspond to similar patterns found in the domain of modal judgments. Adults, it seems, have two notions of modality at their disposal: a more primitive (ontogenetically old) default notion that does not differentiate between descriptive and normative modals and thus yield characteristic signature limits; and more differentiated and nuanced notions (ontogenetically more recent) that do sharply distinguish between different forms of modality. What works fast and gets addressed in speeded tasks is the primitive default notion (in speeded tasks, adults tend to confuse what is possible with what is permitted, for example, in the way very young children do) whereas the more nuanced notions reveal themselves in reflective task settings in which adults are not subject to such confusions (Phillips and Cushman, 2017). Modality judgments and factive ToM may thus reveal striking analogies. Just like in the area of modality, then, there may be basic and default factive ToM, present from early on and in operation throughout the lifespan in speeded responses (and under other conditions of limited cognitive resources), and more sophisticated factive Theory of Mind that develops later on the basis of full-fledged meta-representation and that reveals itself in more reflective judgements.

## Author contributions

Both authors contributed to the idea and conception of the commentary. HR wrote the first draft of the manuscript. Both authors revised the manuscript, read, and approved the submitted version.

## Funding

This work was funded by the Deutsche Forschungsgemeinschaft (DFG, German Research Foundation)—RA 2155/7-1.

## Conflict of interest

The authors declare that the research was conducted in the absence of any commercial or financial relationships that could be construed as a potential conflict of interest.

## Publisher's note

All claims expressed in this article are solely those of the authors and do not necessarily represent those of their affiliated organizations, or those of the publisher, the editors and the reviewers. Any product that may be evaluated in this article, or claim that may be made by its manufacturer, is not guaranteed or endorsed by the publisher.
