# Corrigendum: Knowledge before belief ascription? Yes and no (depending on the type of “knowledge” under consideration)

**DOI:** 10.3389/fpsyg.2022.1055960

**Published:** 2022-10-11

**Authors:** Hannes Rakoczy, Marina Proft

**Affiliations:** Department of Developmental Psychology, University of Göttingen, Göttingen, Germany

**Keywords:** knowledge, belief, factive, Theory of Mind, aspectuality, mental states

In the published article, there was an error in the **Funding** statement. We forgot to mention funding by the “Deutsche Forschungsgemeinschaft (DFG, German Research Foundation).” The corrected funding statement appears below.

## Funding

This work was funded by the Deutsche Forschungsgemeinschaft (DFG, German Research Foundation)—RA 2155/7-1.

The authors apologize for this error and state that this does not change the scientific conclusions of the article in any way. The original article has been updated.

## Publisher's note

All claims expressed in this article are solely those of the authors and do not necessarily represent those of their affiliated organizations, or those of the publisher, the editors and the reviewers. Any product that may be evaluated in this article, or claim that may be made by its manufacturer, is not guaranteed or endorsed by the publisher.

